# Experimental and Finite Element Investigations of Damage Resistance in Biomimetic Composite Sandwich T-Joints

**DOI:** 10.3390/ma9070510

**Published:** 2016-06-24

**Authors:** Ali A. Saeid, Steven L. Donaldson

**Affiliations:** 1 Department of Mechanical and Aerospace Engineering, University of Dayton, 300 College Park, Dayton, OH 45469-0243, USA; saeida1@udayton.edu; 2 Department of Civil and Environmental Engineering and Engineering Mechanics, University of Dayton, 300 College Park, Dayton, OH 45469-0243, USA

**Keywords:** composite sandwich T-joints, biomimetic approach, multiple delamination, bending strength, cohesive zone method, fracture modes

## Abstract

Composite sandwich structural joints, such as T-joints, are used in many different composite applications to transfers the load orthogonally between two sandwich elements. However, these joints connecting the sections can represent the weakest link in sandwich composite structures due to the lack of reinforcement in the out-of-plane direction. Therefore, this paper presents a new methodology for the design and analysis of composite sandwich T-joints using new biomimetic fabrication methods. The fabricated idea comes from biological fixed joints as an evolutionary alteration processes of trunk-branches of trees. It offers unique attributes to optimize the continuous fiber paths for minimum stress concentrations and multi-sandwich layers to increase the bending stiffness and strength. The focus is on how the biomimetic technique can improve sandwich T-joint structures by increasing their strength and load carrying capability without adding a significant weight penalty. The major attention is to investigate the comprehensive failure modes in the joint numerically and verified by experiments. Investigations were conducted on three different designs of biomimetic composite sandwich T-joints under tension and bending loads. The results show significant improvements to the ultimate load up to 68% in the case of bending load and 40% in the case of pull-off load in the biomimetic sandwich T-joints compared to the reference conventional T-joint design. The final failure was significantly deferred in both load status. The FE models provided important insights into the core failure and delamination of multi-interface biomimetic T-joints.

## 1. Introduction

In aerospace, marine and other applications, composite sandwich structures are used extensively. This is primarily due to their low weight combined with high bending stiffness and strength. Several types of joints are fabricated with sandwich panels, including adhesively-bonded and bolted joints. The advantage of adhesively-bonded joints over the bolted joint is that the use of fastener holes in mechanical joints inherently results in micro and local damage to composite laminate during its fabrication, and it would reduce the strength of the joint [1]. However, the main issue in a bonded joint is developing a safe design that can resist through-thickness stresses across the adhesive layers. The major weakness is represented in facesheet composite material, which has low interlaminar toughness due to the low ductility and strength because of the polymer matrix phase. Add to that, in core materials, the shear failure influences the joint structure integrity by reducing the stiffness and strength of the sandwich structure; therefore, it needs to be taken into consideration for sandwich structure design. These issues are deeply the concerns with composite sandwich structural joints and stiffened panels, which are used extensively throughout the air-space and marine frames.

One of the most common adhesive joints between sandwich panels is a composite sandwich T-joint. The method of fabrication of a sandwich T-joint could provide high strength to delay the damage initiation in core and skins with the efficient transfer of the load between the components when the joint is exposed to the different load conditions. The basic design of a composite sandwich T-joint consists of core sandwich panels (web and flange) joined by the overlaminate forming the gap of the fillet. When those components are bonded, the performance of the joint can tolerate the load bearing capacity by transferring the load path between the web and flange. In addition, the shape of the formed fillet could improve the joint performance when it distributes the stresses efficiently around the fillet. The fillet gaps are filled with a polymer adhesive in the conventional design; however, foam fillets, such as triangular and circular poly(vinyl chloride) foam fillets, have been used in improved design studies [2,3]. It was found that the T-joint with triangle fillets provides 20% higher strength than the circular one, with the obtained a weight reduction of 60%. Numerically, the effect of fillet geometry and core material types was investigated, and it was found that the best base angle of a triangle fillet was 45 degrees; and the joint failure load changed by the changing of core types [4]. Meanwhile, the two main failure modes, which were the interfacial debonding failure between the fillets and facesheet overlaminate and the shear failure in the core flange, were also observed.

Other geometry effects on the strength performance of a composite T-joint have been investigated experimentally and numerically. In particular, the influences of overlaminate thickness (number of facesheet layers) and fillet radius size (length of lap) were described on the relevant stress components [5]. It was shown that increasing the attachment length and thickness had the effect of delaying the onset of interface failure. However, the geometry and stacking sequence of overlaminate had no effect on the failure location [6,7]. Despite the fact that increasing the radius of the fillet could raise the possibility of crack initiation on the fillet region since it would be a rich resin area, this can be considered another failure mode of T-joints. Overall, the composite T-joints undergo different failure modes. It depends on the load conditions, the lay-up sequences and the geometrical shapes of the components. Subsequently, improving the failure strength and mechanical performance of the sandwich T-joint can be achieved by several ways. Accordingly, experimental and numerical studies of fabricated sandwich T-joints using z-fiber insertions in the flange interface and directly in the radius filler show increased initial and ultimate tension failure load with additional z-pin insertions. The reason for that is to resist crack growth along skin-core interface in the curvature area, as it represents the weakest area of delamination/debonding [8,9].

Another design development of the carbon fiber/epoxy T-joint was investigated by fabricating the T-joint with the embedded structural feature of the tree joint, dropping off the lay-up from the stiffener to be terminated in the flange, which carried a higher load, as well as increasing the inelastic strain energy and absorbed strain energy, but the expense of the earlier onset of damage initiation was gained [10]. Thummalapalli and Donaldson suggested a new sandwich T-joint based on biomimetic concepts, which had the characteristics of a non-circular fillet, continuous fiber from flange to web and the use of multi-layer cores. They examined the joint under only the bending load condition and found that the ultimate failure strength increased up to 39% over the conventional design [11]. However, among the investigations of the biomimetic hypotheses’ design idea on the sandwich composite T-joint, in the absence of testing under different load conditions, numerical studies, such as the adhesive failure of initiation and the propagation of cracks to cause the separation of joint elements and core shear failure, are highlighted.

The complicated geometry of T-joints can be designed to improve the failure strength and mechanical performance of the sandwich T-joint as it reduces the induced interlaminar stresses in critical regions. The study by Mattheck and Bethge [12] found that a biomimetic approach can be used as an optimizing technique to achieve uniform stresses across the joint, such as wood and bone. Subsequently, the biomimetic method is considered to fabricate composite sandwich T-joints to enhance the strength of the joint. It could delay the damage initiation in the core and skins to maximize the efficiency of transferring the load between the components. The main advantage of this technique is that the fabrication technique of the sandwich T-joint would eliminate specific high stress sites when the failure is initiated, as well as maximizing the delay of final failure.

Therefore, in the present work, the main objectives were to investigate two new sandwich T-joint designs based on biomimetic concepts experimentally and numerically. The new T-joints designs were formed with continuous fiberglass as a skin from flange to web and used the multi-layer of cores to simplify the fabrication, which additionally saved weight and enhanced the stiffness of the T-joint. The two biomimetic T-joints were designed to simulate the shape area of the connection between branch to trunk as a v-notched shape. The mechanical testing from bending to pull-off loads was conducted to examine the stiffness/strength and the performance of joint efficiency.

## 2. Biomimetic Approach for Designing T-Joint Geometry

**Figure 1:**
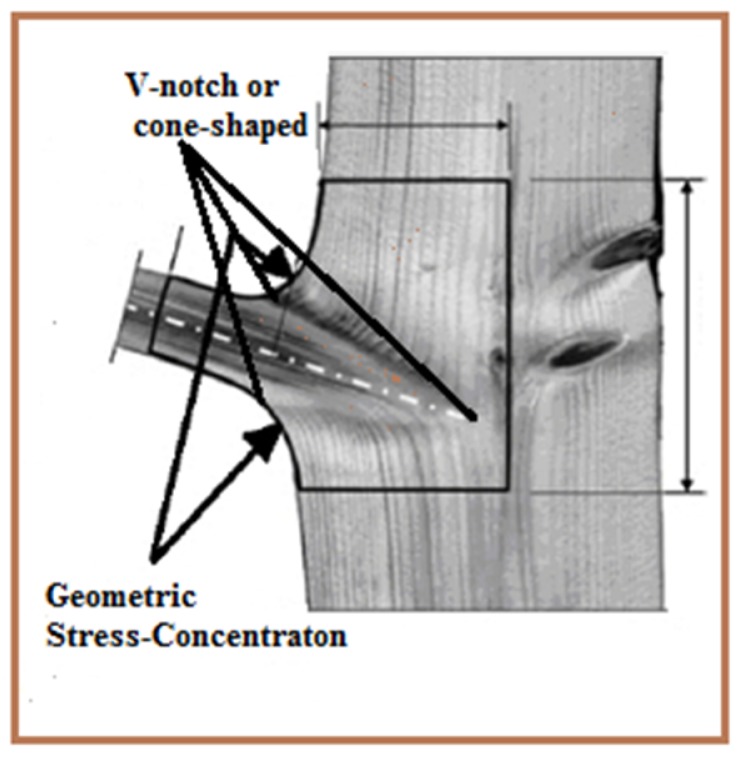
Branch-trunk connection showing v-notched and geometric stress concentration areas [13].

The design principle of biomimetic fabrication is to apply the self-optimized process observed within biological structures from trunks to the branches of trees [11,12]. As in a tree, as in Figure 1, the density and orientation of the wood fibrils between branches to the trunk are optimized by the evolutionary process to achieve uniform strain conditions across the joint, even though the geometry of the tree joint has a high stress concentration in the shape area of the connection between branches to the trunk. Additionally, the advantage of uniform strain is that overload can be distributed in such way as to make the weak sites in the joint have less effects from cracks and delamination, and therefore, the structural performance efficiency of the joint is maximized [13].

Therefore, the special characteristics of the connection between branch to trunk are applied in the T-joint geometry. Reducing the stress concentration in the critical area of the joint was the main design target. The two main wood fibril attributes are the continuity of fibrils from trunk to the branch with the ability of altering the orientation and the densification of fibril stacking to each other to form the heart wood. These attributes can be represented in composite sandwich T-joint geometry as the continuous fiber from flange to web with an altering fiber layup direction and using a multi-layer core with different thickness to make robust the densification with the advantages of being lightweight and energy absorption.

**Figure 2:**
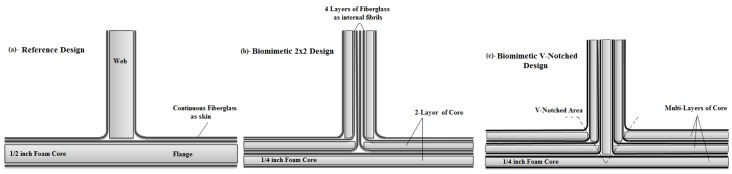
Strategic fabrications of the structural configurations of T-joints. (**a**) Reference design; (**b**) biomimetic (2 × 2); (**c**) biomimetic v-notched (3 × 3).

In this study, the basic design of the sandwich T-joint was fabricated as the reference T-joint design with only continuous fiber to connect the flange (base) to the web (stiffer), as in Figure 2a. It can be divided into four main parts: a half inch-thick layer of core as the flange, a another core layer as the web, continuous fiberglass as the skin and the filler from epoxy, where the four parts are connected to each other with bonding lines. Figure 2b shows the biomimetic T-joint design. The biomimetic approach was applied in which the thickness of core layers in the flange and web were divided from half inch into two quarters of inch, as a thin core to facilitate the continuous fiberglass to run though. The target in this design was to stiffer the web where four layers of fiberglass stacking together in the middle of the web. This design could reduce the stress concentration in the filler region area because of the reinforced web, resulting in the delay of the final failure; however, it makes the filler region larger, so as to be more prone in the resin-rich area, causing the matrix to crack.

Subsequently, the v-notch shape in the connection between the trunk and branch was considered to restrict the rich resin zone area to eliminate the crack growth. It can make the design more robust, because the most growing crack occurs in that area, which represents the rich resin area in the T-joint [14]. Therefore, it was applied to the T-joint design, as in Figure 2c. In this design, multi-core layers were used to make the v-shape. The continuous fiberglass was distributed in such way so as to achieve a uniform stress condition across the joint. Additionally, the advantage of this is to make the overload in weak sites distributed across the joint to delay the cracks and prevent materials from losing stiffness, and therefore, the structural efficiency of the joint is maximized.

**Figure 3:**
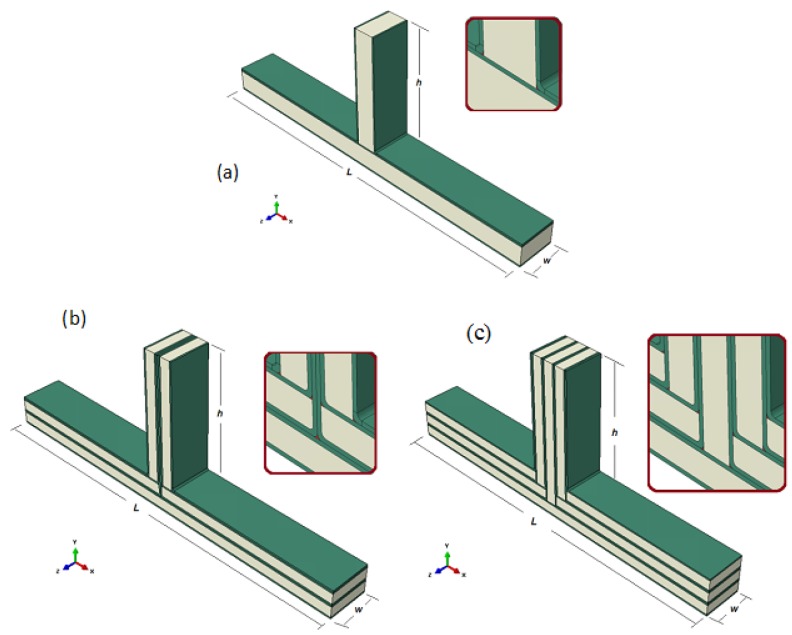
Geometric details of T-joint configurations. (**a**) Reference design; (**b**) biomimetic (2 × 2); (**c**) biomimetic v-notched (3 × 3).

Furthermore, the three sandwich composite T-joint geometries were fabricated to be tested in a series of experiments. The bending and pull-off loading were applied on the T-joints to investigate delamination/debonding and material degradation effects on the performance of the joint. This would explore the biomimetic design idea to highlight the outline of the design principle of the joint. Figure 3 and Table 1 present the detailed geometry of the fabrication for the reference, biomimetic II and biomimetic v-notched composite T-joints. Note that L and W are the total length of the joint and the width of the joint, respectively, while h is the height of the web measured from the top skin on the flange.

## 3. Biomimetic Composite Sandwich T-Joint Fabrications

The sandwich composite T-joints were fabricated based on the cross-sections of the reference design and each new fabricated design of the biomimetic T-joints given in Figure 3. The T-joint consists of a web and a flange cut from a Divinycell foam H80 core panel [15] and adhesively bonded with a continuous single layer of S-1 glass unidirectional fiberglass [16]. The T-joint specimens were prepared in accordance with the dimensions given in Table 1. The epoxy adhesive was used to fill the filler regions, and it was the EPON 828 resin epoxy well mixed by a ratio of 10:1 parts with EPI-CURE 3223 hardener as the curing agent [17].

**Figure 4:**
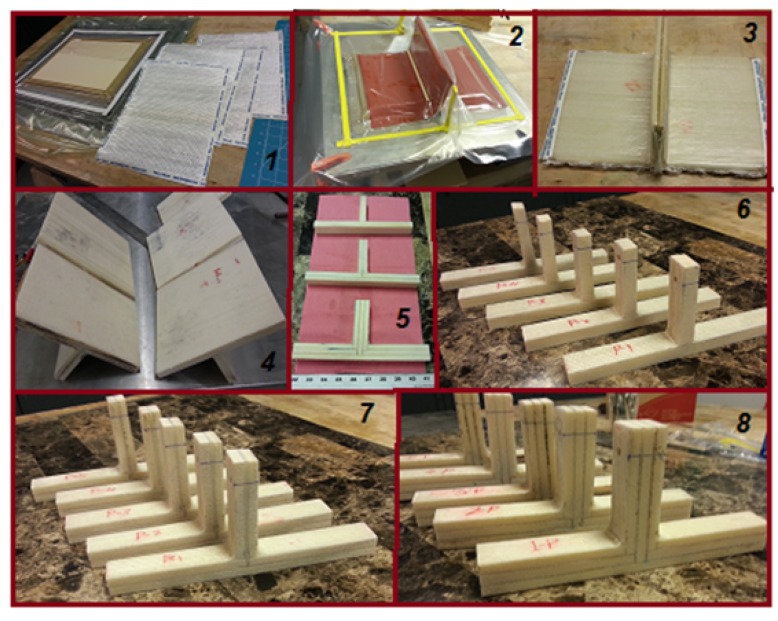
(1) Cut foam and fiber; (2) vacuum bag set-up; (3) specimen after a cure; (4) final cured specimen; (5) cutting of the finished specimen; (6) samples of the T-joint reference design; (7) samples of the T-joint biomimetic design; (8) samples of T-joint v-notched biomimetic design.

The structural complexity of the biomimetic T-joint geometry requires a suitable process of fabrication to achieve the design target. Figure 4 shows the details of the processes to gain the maximum performance of the designed T-joint. One such technique is how to make the vacuum bag distribute the pressure equally to all surfaces of the T-joint. This was taken into consideration because the T-joint has an angle on both sides, and therefore, the pressure is generated gradually until the resin expelled uniformly between the web/flange core and the fiberglass laminates.

The first step was to cut the foam H80 sheets to the flange base dimensions of (12 inches ≈ 30.5 cm) long and (9 inches ≈ 23 cm) in width and the web core shape of (5 inches ≈ 12.7 cm) in height and (9 inches ≈ 23 cm) in width for both sheets with a thickness of (0.5 inches ≈ 1.27 cm and 0.25 inches ≈ 0.635 cm). The fiberglass was cut to be consistent with the foam dimensions, as in Figure 4(1). For the process of fabrication, the work surface plate was cleaned; then, the tape dummy was placed in such way to make the resin well spread. By following the suggested design shape of the biomimetic T-joints, each design configuration was built with care in a short time because of the resin;s limited cure time. The thick rubber sheet of 1.5875 mm (1/16 of an inch) was placed on the surface of the T-joint inside the vacuum bag to create more pressure to bond the fiberglass and core well, in addition to making the final surface smoother. For making the angle of the connection of the flange with the web of the required dimensions, two circular wood bars were placed above the rubber sheet, close to the angles. After that, the vacuum bag was set up to make the vacuum machine pull the air bubbles mixed with the remaining resin from the middle area of the T-joint, since that place had the most gaps between the parts, as in Figure 4(2). In this process, a pressure of 100 KPa (14.5 PSI) was generated step by step to distribute the pressure uniformly and to make the T-shape consistent with the design requirements. The vacuum bag T-joint was cured for 24 h at room temperature; then, the T-joint specimens were put in a dry place for two weeks for final curing, as in Figure 4(3).

The specimens were cut to the final design shapes for samples of T-joints as the reference design, as in Figure 4(6); and the samples of the T-joint biomimetic 2 × 2 design, as in Figure 4(7); then, the final samples of the T-joint v-notched biomimetic design, as in Figure 4(8). Note that the T-joint was called the biomimetic 2 × 2 joint indicating, the number of the two foam layers in the web and flange, while the T-joint v-notched biomimetic design indicated the v-shaped design. Clearly, it seems that the level of structural complexity increases, as well as the amount of continuous fibers passing from the flange to web between the biomimetic T-shapes as the more advanced designs.

## 4. Mechanical Test Configurations and Experimental Procedure

The quasi-static tests were applied in bending and pull-off loading configurations for all three fabricated T-joint designs after applying the biomimetic design concepts. The goal of the project was to examine how the two new biomimetic T-joint designs delay the damage initiation and enhance the final strength of the joint up to failure compared to the reference T-joint design. The details of the test procedure for bending and pull-off tests are shown as follows.

### 4.1. Procedure of Bending/Compression Test Configuration

**Figure 5:**
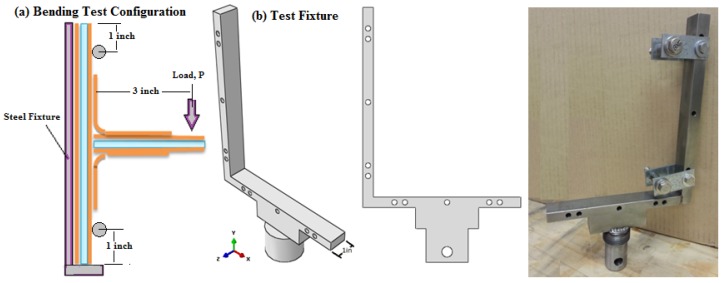
(**a**) Bending test configuration; (**b**) fixture of the T-joint bending test.

The T-joint bending test was conducted using an INSTRON 4486 universal testing machine at a cross-head displacement rate of 1 mm/min (0.03937 inches/min). The details of the boundary conditions for the bending specimens’ set-up and the used fixture can be seen in Figure 5. The bending test was performed to apply the load as a line of static compressive load on the top of the web, which acts along the depth of the specimen and parallel to the machine frame aligned in the center of the fixture. For the reference T-joint specimens, the test was terminated once the load dropped to half of the peak force, while the test was forced to stop after exceeding the displacement of 20 mm in both new biomimetic T-joints. The data were recorded and displayed as the compression force/displacement for all three T-joint samples.

### 4.2. Procedure of the Pull-Off/Tension Test Configuration

**Figure 6:**
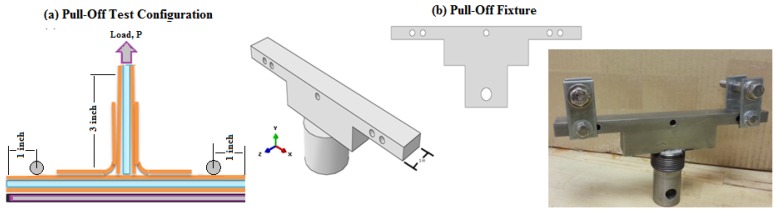
(**a**) Pull-off test configuration; (**b**) fixture of the test.

The pull-off test was conducted to examine the three T-joints in tension using an INSTRON 4486 universal testing machine at a cross-head displacement rate of 1 mm/min (0.03937 inches/min). The pull-off test was performed to pull-out the load from the loading fixture (pin) acting on a drilled hole of 8 mm in diameter of the two (2-inch length × 1-inch width) aluminum plates fixed from the lower side with the web section with an 8-mm bolt/nut. For the details of the test conditions for the specimen set-up and the fixture that was specifically designed, see Figure 6. Five specimens of each T-joint design were tested in which the test was terminated automatically once the load dropped to half of the peak force in the reference design; in contrast, the test was forced to stop after exceeding the displacement of 30 mm in both new biomimetic T-joint designs. The test data were recorded and displayed as the tension force/displacement curves for all three T-joint designs.

## 5. Damage Resistance Modeling in the Biomimetic Sandwich T-Joint

**Figure 7:**
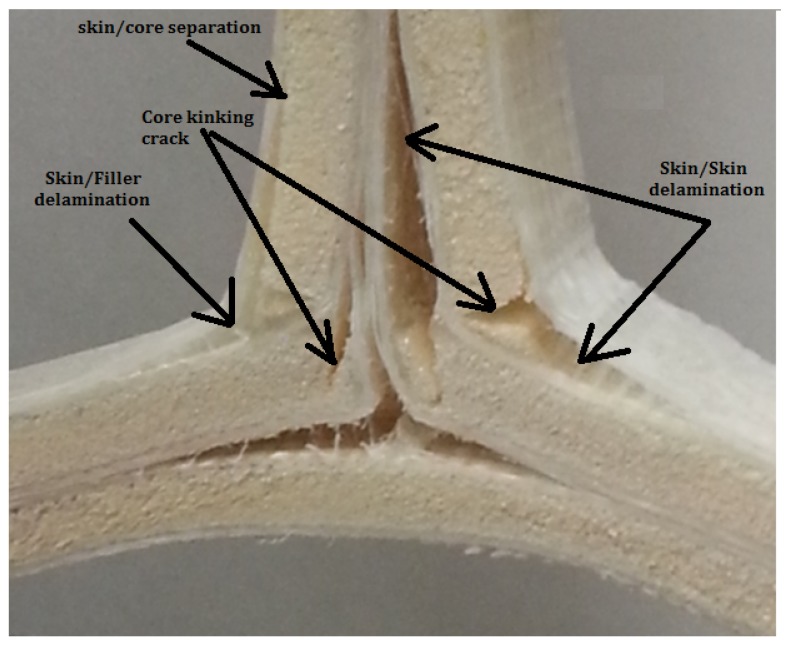
Summary of the experimental failure modes.

In finite element investigations, the main target of modeling the sandwich biomimetic T-joint damage is to investigate the delamination/debonding and material degradation effects on T-joint strength performance through loading angles from 0° (tension pull-off load) to 90° (bending load). In this research, considerable failure criteria for modeling damage are assigned to each T-joint model based on the major failure mechanisms observed in T-joint experiments, as in Figure 7. It is clear that the location of failure is dependent on the geometric and material parameters. The sequence of failure events can be described by the first failure mechanism, which is the debonding of the skin/filler interface occurring in the T-joint curvature. The second failure mechanism is the initiation by the kinking crack in the core material for some areas in the flange and web components. The third failure mechanism is the delamination between the fiberglass plies. The fourth failure mechanism is the failure initiation by the debonding of the skin/core in the vertical direction. The main reason for the delamination is the interlaminar normal and shear stresses between different constituents of T-joint components, while the failure initiation of the kinking crack could be caused by the core shear failure.

Subsequently, the commercial finite element package Abaqus/Standard is used to simulate the mechanisms of damage response of composite sandwich T-joints under quasi-static loading. The Cohesive Zone Elements (CZM) with zero-thickness under the bilinear cohesive law are used to simulate delamination/debonding at the skin/core, skin/skin and skin/fillet interfaces through the bonding lines in the T-joint structure. The cohesive element has the ability to capture the first failure, and the design parameters of the interface properties are well defined. For modeling the crack kinking damage, the enrichment element is used with the extended finite element combined with the maximum stress criteria to capture the initiation of the crack in the core foam materials. Beside that, three material modes, the elastic-plastic model for core foam, the elastic-brittle mode for skin (fiberglass) and the interface materials, are involved in the T-joint design. All of these parameters are incorporated into each biomimetic T-joint design, after applying the actual size dimensions, to reduce the diversions from the experimental results.

### 5.1. Geometry and Material Modeling

The composite sandwich T-joint geometries were modeled in two dimensions. The size of each T-shape was taken based on the experimental T-joint shape before each T-joint design was tested in a series of experiments under bending and pull-off loads. The geometrical details of the modeling for the reference, biomimetic II and biomimetic v-notched composite T-joint are as in Figure 2 and Figure 3 and Table 1.

All three T-joint designs were modeled with the elastic orthotropic properties of the composite material for S1-glass/epoxy, while the core material of foam H80 and adhesive material of EPON/epoxy for the filler were modeled as isotropic elastic properties. The elastic material properties are presented in Table 2. For the plasticity of the core foam material, it was modeled using the Abaqus implemented Isotopic Crushable Hardening (ICH) model with input parameters for the in-plane tension parameters reported in [18]. For the brittle response of the fiberglass, it was modeled with the Hashin damage model in which the damage initiation parameters had the strength properties of fiberglass, as in Table 2, while the evaluation of the damage parameters is calculated based on the element characteristic length of $L^{c}$= $9.77\times 10^{-4}$ m, as shown in Table 3.

**Figure 8:**
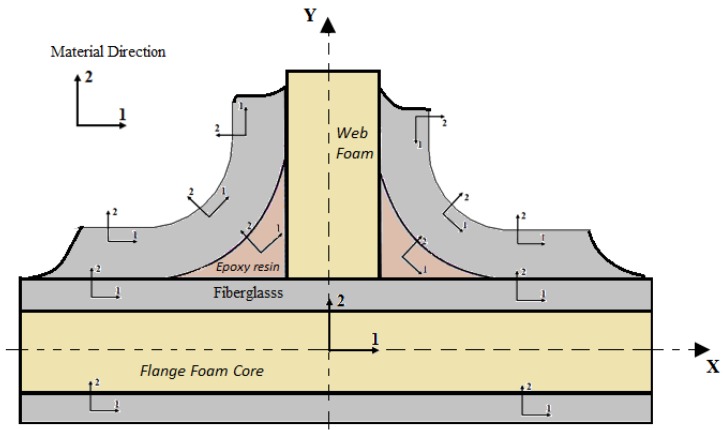
Local material directions.

Figure 8 shows the local material directions used in the modeling of all sandwich composite T-joint designations. For the skin of composite sections, the first direction is the fiber (0°) direction, and the second direction is the through thickness (90°) direction. The cylindrical coordinate directions are applied on both the fiber curvatures and sides of the filler section to make the stress distribution similar at the curved region. For cohesive elements, the second direction represents the opening-mode direction having one element, while the first direction represents the shear-mode direction. The core material directions are ignored because they are modeled as an isotropic material.

**Figure 9:**
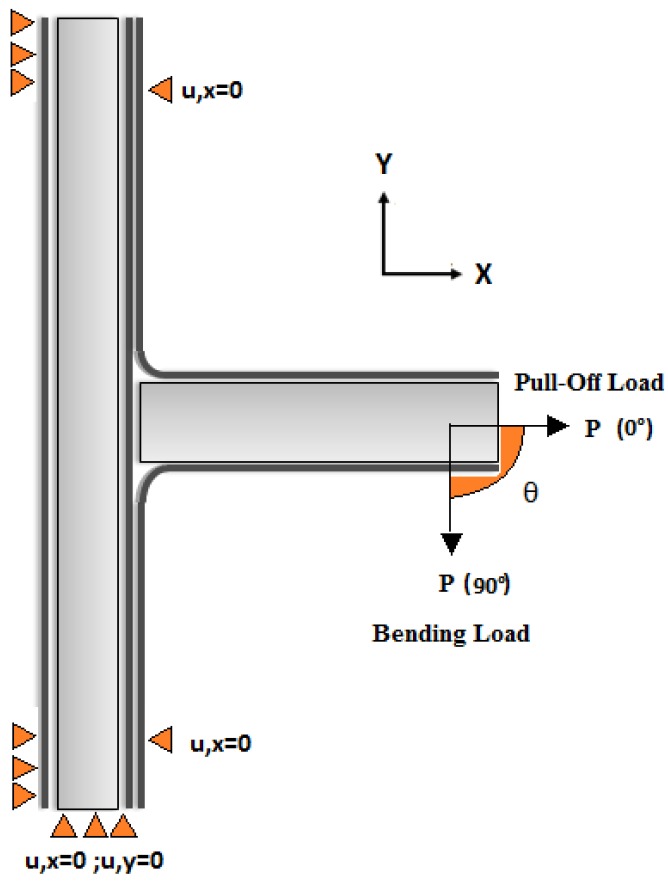
The loading and boundary conditions for angle pull loads.

To validate the experimental results, the angle loads at 0° as the pull-off load and 90° as the bending load are used to characterize the three T-joint designs for damage modeling of interfacial delamination and core kinking failures. Figure 9 shows the loading and boundary conditions for T-joint tests. After the validation with the experimental results in the load applied to 0° as the pull-off load and 90° as the bending load, the mode of the T-joint will be used to test the T-joints in which the load will be applied by angles of 15°, 30°, 45°, 60° and 75° measured from the *y*-axis. This would examine the comprehensive failure range of the T-joint structure under the biomimetic design hypotheses for stiffness investigation.

### 5.2. Mixed-Mode Damage of the Biomimetic T-Joint

**Figure 10:**
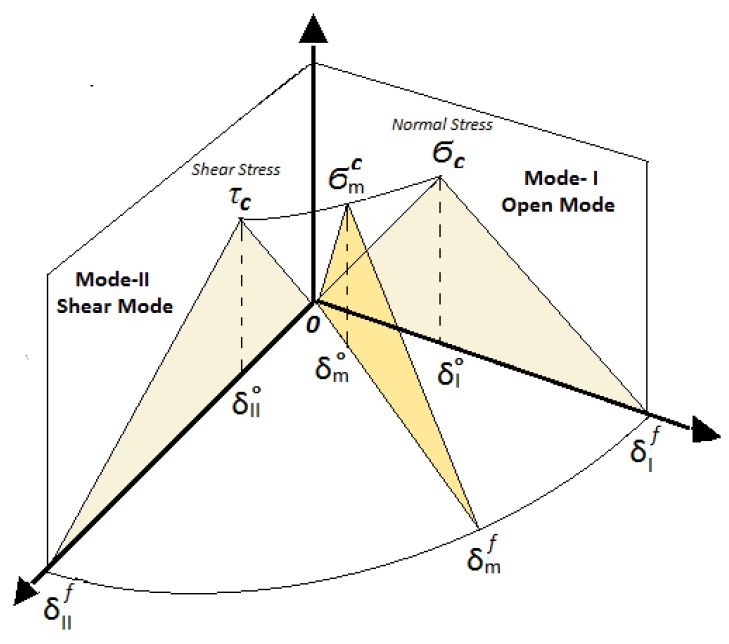
Sketch of the mixed-mode bilinear cohesive law [23].

In the experimental tests of the sandwich composite T-joint structures, the delamination growth occurs most certainly under mixed-mode failure loads. Subsequently, a general formulation for the cohesive element used in the onset and propagation of mixed-mode delamination is required to investigate the interface properties. The aim of this is to understand the failure sequence of such multi-hybrid-interface structures. This can be achieved by identifying the mixed-mode bilinear cohesive law of each interface represented as the orange triangle of points (o, $\sigma_{m}^{c}$ and $\delta_{m}^{f}$), as in Figure 10. To start with, it is assumed that all of the damages are occurring under the combination of Mode-I and Mode-II, since the T-joint modeling will be constructed only in two dimensions. The onset of damage can be predicted using the quadratic failure criterion [21,22].

(1) $${\left(\frac{\langle \sigma \rangle }{\sigma_{c}}\right)}^{2}+{\left(\frac{\tau }{\tau_{c}}\right)}^{2}\geq 1$$

where *σ* and *τ* are the normal and shear stresses at an arbitrary point. Based on the criteria, the damage in the cohesive element is assumed to start when the left part of Equation (1) is equal to or bigger than one. At that point, the *σ* and *τ* will have the same values, called the critical stress of traction for mixed-mode damage ($\sigma_{m}^{c}$), as in Figure 10. Corresponding to that, the initial separation will have the initial mixed-mode relative displacement ($\delta_{m}{}^{\circ}$) that can be calculated from the mixed-mode relative displacement at any arbitrary point, which was defined [23] as:

(2) $$\delta_{m}=\sqrt{\langle \delta_{Normal}^{2}\rangle +\delta_{Shear}^{2}}$$

The initial displacement of opening and shear modes can be calculated before softening onset using the penalty stiffness and the traction of each mode as:

(3) $$\delta_{I}^{{}^{\circ}}=\frac{\sigma_{Normal}}{K_{I}}$$

(4) $$\delta_{II}^{{}^{\circ}}=\frac{\tau_{shear}}{K_{II}}$$

Then, the initial mixed-mode relative displacement corresponding to the onset of softening is:

(5) $$\delta_{m}^{{}^{\circ}}=\delta_{I}^{{}^{\circ}}\delta_{II}^{{}^{\circ}}\sqrt{\frac{1+\beta^{2}}{\delta {{}_{II}^{{}^{\circ}}}^{2}+\delta {{}_{I}^{{}^{\circ}}}^{2}\beta^{2}}}$$

where *β* is defined as the mixed mode ratio as $\beta =\delta_{II}^{{}^{\circ}}/\delta_{I}^{{}^{\circ}}$.

To predict the delamination propagation, Benzeggagh–Kenane criteria are used under mixed-mode loading conditions [21]. It is established in terms of the energy release rate of Mode-I and Mode-II and the mixed-mode ratio of fracture toughness. It is also one of the most used propagation laws and implemented in Abaqus as given as:

(6) $$G_{IC}+\left(G_{IIC}-G_{IC}\right){\left(\frac{G_{IIC}}{G_{T}}\right)}^{\eta }\leq G_{C}$$

where $G_{C}$ is the total fracture toughness for a specified mixed-mode ratio and *η* is the BKlaw exponent extracted from the experimental results by curve fitting. By using the BK law, the final critical relative displacement for the mixed-mode can be calculated as [23,24]:

(7) $$\delta_{m}^{f}=\frac{1}{K_{i}^{{}^{\circ}}\delta_{m}^{{}^{\circ}}}\left[G_{IC}+\left(G_{IIC}-G_{IC}\right){\left(\frac{\beta^{2}}{1+\beta^{2}}\right)}^{\eta }\right]$$

After determining the parameters of the traction-separation of the mixed modes, the mixed-mode damage parameter can be calculated as [21]:

(8) $$D=\frac{\delta_{m}^{f}\left(\delta_{m}-\delta_{m}^{{}^{\circ}}\right)}{\delta_{m}\left(\delta_{m}^{f}-\delta_{m}^{{}^{\circ}}\right)}$$

where $\delta_{m}$ is the crack propagation displacement for the mixed-mode at any arbitrary point. The mixed-mode damage parameter (D) is equal to zero at the onset of damage ($\delta_{m}^{{}^{\circ}}$) and one at the end of the delamination stage ($\delta_{m}^{f}$).

**Figure 11:**
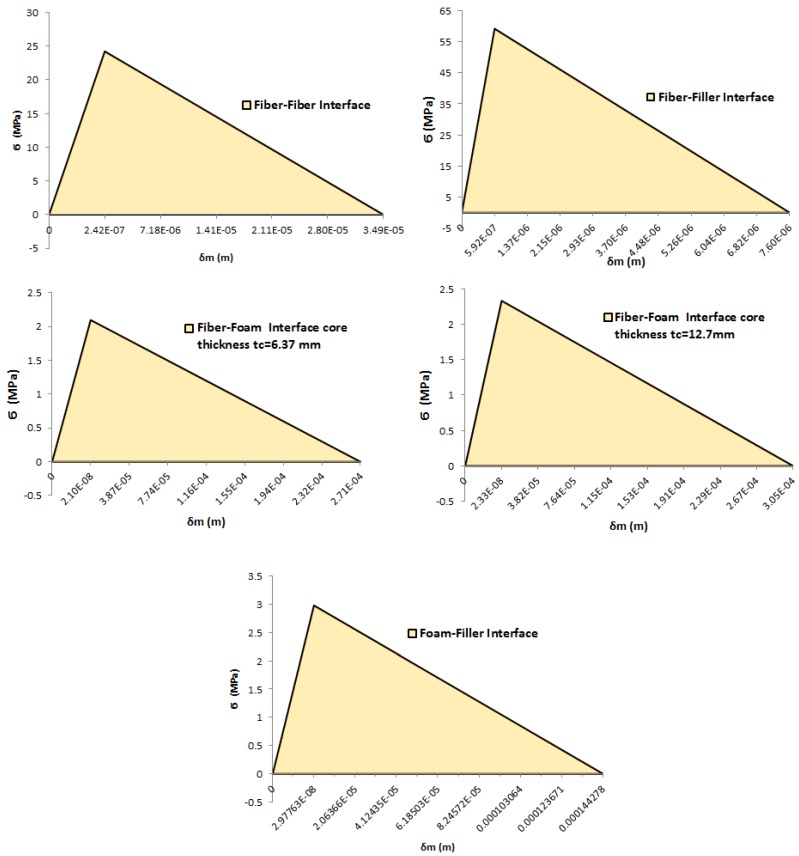
Mixed-mode of stress and *δ* values for T-joint material interfaces.

Ultimately, Figure 11 shows the mixed-mode traction-separation triangles of the interface properties used of the mixed-mode damage of the biomimetic T-joint modeling designations. Each interface triangle was calculated using Equation (1), Equation (5), Equation (7) and Equation (8) and the properties of that bonding line, as in Table 4, for the interfaces of the fiber/fiber, fiber/filler, foam/filler and two different interfaces of the foam/fiber based on the thickness of the core materials.

The basic idea to use this technique is to modified the cohesive parameters, i.e., the cohesive penalty stiffness parameters ($K_{I,II}$) and the power BK law exponent (*η*), and the others parameters are fixed. Since the penalty stiffness parameters are a function of the normal and shear stresses, as in Equation (3) and Equation (4), they can change the stresses to have the same value in Equation (1), resulting in the critical mixed-mode stress ($\sigma_{m}^{c}$); at that point, the cohesive penalty stiffness parameters ($K_{I,II}$) are recorded for that bonding line. For the second parameter, the power BK law exponent (*η*), it can change the final critical relative displacement for the mixed-mode based on the length of the cohesive element of each mode (using 0.001 mm in our modeling), to make the final mixed-mode damage parameter (D) equal to one. After calculating the ($\delta_{m}^{f}$) for the initial value of (*η*), using Equation (7), the end of the triangle may not end in the correct position; thus, by changing the (*η*) parameter until the triangle ends became identical, at that point, the (*η*) is recorded for that interface.

Clearly, Figure 11 of the mixed-mode CZM triangles confirms that the first failure occurs in the fiber/filler interface, since it has the highest critical traction mixed-mode stress ($\sigma_{m}^{c}$) and the lowest final separation relative mixed-mode displacement ($\delta_{m}^{f}$). The second interface of first failure will be in the fiber/fiber interface, then the foam/filler interface next and, lastly, the fiber/foam interfaces. This helps us to overcome some of the modeling contact issues and to gain the logical modeling results.

### 5.3. Finite Element Model of the T-Joint under Bending and Pull-Off Loading

In this section, the commercial finite element package Abaqus/Standard (v6.13-3) [21] was utilized to simulate the mechanical response of sandwich composite T-joints under quasi-static bending and pull-off load. The three T-joint geometries are modeled in 2D based on the dimensions of the experiment shapes, while the thickness of the T-joint is assigned from the section panel. The boundary conditions of the bending test are similar to the experiment of each T-joint design, as shown in Figure 9. The bending load is attached to the upper facesheet of the web section laying in the Y-direction as the prescribed displacement, while the down end of flange is fixed, and the upper flange able to move in the Y-direction. For the pull-off load, it is applied to the reference and biomimetic T-joint models as a symmetric condition in which half of each model has been taken into the analysis with the applied symmetric boundary conditions.

An FE model of each T-joint design was constructed with the plane stress assumption for a composite facesheet skin, since the Hashin failure criteria only worked with plan stress elements. The facesheet skins were modeled with the element type of CPS4I (a four-node bilinear plane stress quadrilateral with incompatible modes). However, the plane strain assumption was used to model the core material, since the crushable foam model worked only with the plane strain elements. The web/flange of the foam core panels and the filler regions were modeled with the element type of CPE4I (a four-node bilinear plane strain quadrilateral with incompatible modes). For materials properties, the anisotropic properties were assigned to the facesheet skins, while the isotropic properties were assigned to the core panels and filler regions, respectively, as in Table 2. The cohesive layers were modeled as interface elements with zero-thickness with the element type of COH2D4 (cohesive elements of 2-dimensional with 4 nodes) between each interface bonding line. The triangular traction-separation cohesive law was used to model the cohesive properties with quadratic stress criteria for delamination initiation and the BK criterion for delamination propagation, as in Table 4.

For mesh details, in the reference T-joint model, the the web/flange core panels had total elements of 27,258 with the element type of CPE4I, and the number of elements through the thickness of the web/flange panels was 21 elements with an aspect ratio of 10:1.7. The filler regions had a total number of 106 elements of the CPE4I type. For skin facesheet layers, they had total elements of 11,088 of type CPS4I, and five elements through the thickness of each layer were used with an aspect ratio of 10:1.9. Regarding the bonding lines, the cohesive element type of COH2D4 was used with total elements of 2307 with one element through the thickness. The total nodes of the FE model were 46,277. In the same technique, the biomimetic (2 × 2) T-joint model had total elements of 45,923 with the element type of CPE4I for core panels, and the number of elements through the thickness was 20 elements for each panel with an aspect ratio of 10:1.12. The skins facesheet layers had a total number of 25,619 of the CPS4I element type with the number of elements through the thickness being five for each layer with an aspect ratio of 10:1.6. The bonding lines used the total of 6217 of the cohesive element type of COH2D4, and the total nodes of the FE mode were 91,321. Lastly, for the biomimetic T-joint v-notched model, it had total elements of 43,196 of CPE4I for core panels, and the number of elements through the thickness was 14 for each panel with an aspect ratio of 10:1.38. The skins facesheet layers had a total number of 26,780 of the CPS4I element type with the number of elements through the thickness of five for each layer with an aspect ratio of 10:1.82. The bonding lines used the cohesive element type of COH2D4 of the total number of 7211; then, the total nodes of the FE mode became 92,651 nodes.

Furthermore, to overcome the numerical issues, a viscosity parameter with a value of 1e-5 is used. The geometrical non-linear analysis, NLGEOM option, is activated, as the deformation of the specimen is expected to have a large opening displacement. The prescribed displacement is applied with a quasi-static step after modifying the line search parameters to make the solutions easily converge for such s non-linear problem. The line search is active only for steps that use the quasi-Newton method. The idea of line search is to correct the solution using a scale factor to reduce the large residuals caused by the cutbacks and sharp discontinuities in the solution. Thus, the line search can be set to iterations ($N^{ls}=10$) and the line search scale factor ($\eta^{ls}=0.01$) to make fewer nonlinear iterations and cutbacks and an overall reduction in solution cost, but the solution may be gained in more line search iterations.

## 6. Results and Discussion

### 6.1. Experimental Results

**Figure 12:**
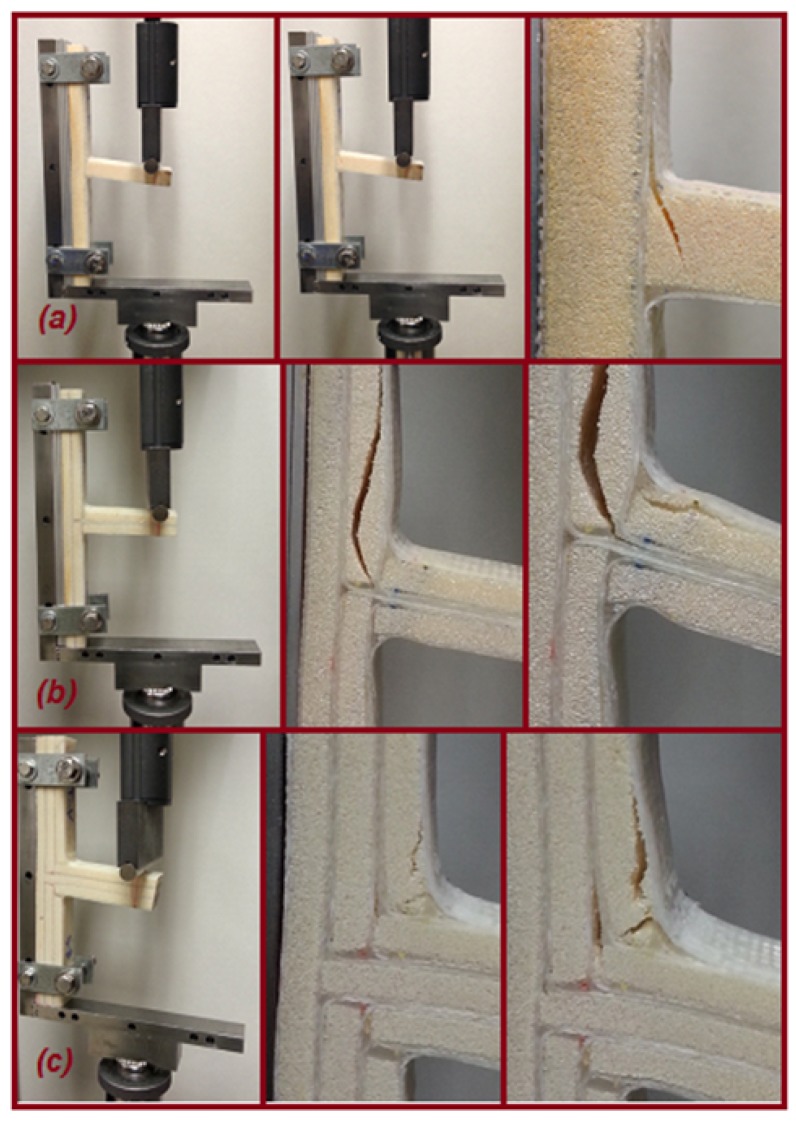
The failure sequence under the bending load of the T-joint. (**a**) Reference design; (**b**) biomimetic (2 × 2) design; (**c**) biomimetic v-notched (3 × 3) design.

**Figure 13:**
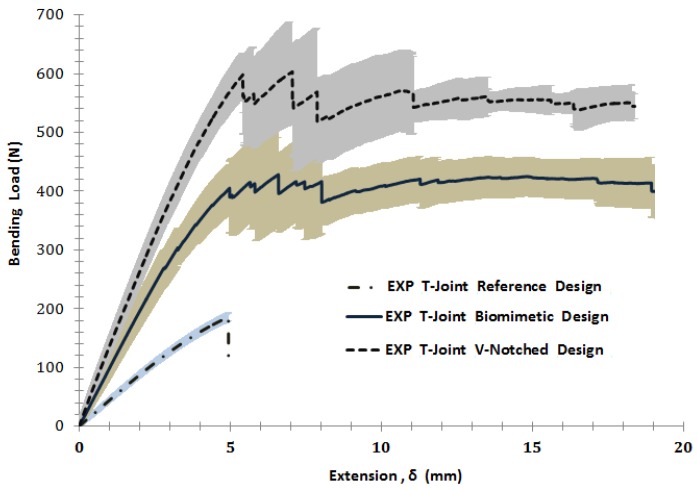
Comparisons of the T-joint strength for the reference design, the biomimetic (2 × 2) design and the biomimetic v-notched (3 × 3) design under bending/compression testing. The lines represent the average values, while the shaded area represents the standard deviation of five specimens of each kind of T-joint design.

Figure 12 and Figure 13 illustrate the comparisons of the failure sequence and the average structural strength response of five T-joint specimens of each T-joint for the reference design, the biomimetic (2 × 2) design and the biomimetic v-notched or (3 × 3) design. As can be seen, when the compression load increased, the reference T-joint samples fail suddenly, as a brittle catastrophic failure at a load of 185 N and a displacement of 4.8 mm. The failure was developed as the crack initiated to cause the debonding between the fiberglass layer and the epoxy filler in the fillet region, then with continuous growing to the web foam core. This indicates that the crack was driven by the mixed mode in the debonding stage, then to the shear failure mode in the web core, as in Figure 12a.

For biomimetic (2 × 2) T-joint samples, it is clear that the strength of the T-joint was increased about two times that of the strength of the reference T-joint design, as in Figure 13. The failure sequence was developed in two area; the debonding was in the fillet region in the middle of the joint when the two major cracks were arrested. They initiated to cause the first failure referred to in the force/displacement graph at a load of 400 N and a displacement of 5 mm. During the load increase, one crack propagated through the upper core flange, and the second grew below the top fillet region of the web, as in Figure 12b. However, the core cracks were not enough to cause earlier failure that destroyed the load bearing. The reason for that was that the reinforcement of four layers of fiberglass that pass through the core flanges are combined together in the central of web core side. This ultimately helped the structure to delay the final failure up to 20 mm of the extension. To conclude, the results show that the biomimetic fabrication enhanced the overall T-joint strength to additionally gain a reasonable delay of the T-joint final failure. Simultaneously, the design maintains the weight to be as close to the reference design weight as possible by making the final biomimetic T-joint thickness equivalent to the thickness of the reference T-joint design.

Furthermore, the result of the biomimetic T-joint with the v-notched design reveals that the joint strength was expanded more than three times that of the strength of the reference T-joint design and 1.5 times that of the strength of the biomimetic (2 × 2) T-joint design, as in Figure 13. This is because of the ability of the imitated v-shape to reduce the interlaminar stress in that critical region. Subsequently, the first failure of the structure was delayed to reach a load of 600 N and an extension of 5.7 mm. However, the failure sequence was the debonding at the upper fillet when the skin separated from the fillet followed by an interfacial crack that propagated up into the foam/fiberglass skin interface area and the kinking crack that grew into the foam core to the center of the joint. When the load increased, another delamination between the skin layers was developed at the area of the combined skin of the upper web/core with the skin of flange/core, as in Figure 12c. To close, the v-notched T-joint was able to sustain the raising of the load to delay the final failure even more compared to the first two cases. The results show the biomimetic v-notched fabrication strengthened the composite sandwich T-joint and effectively postponed the final failure.

**Figure 14:**
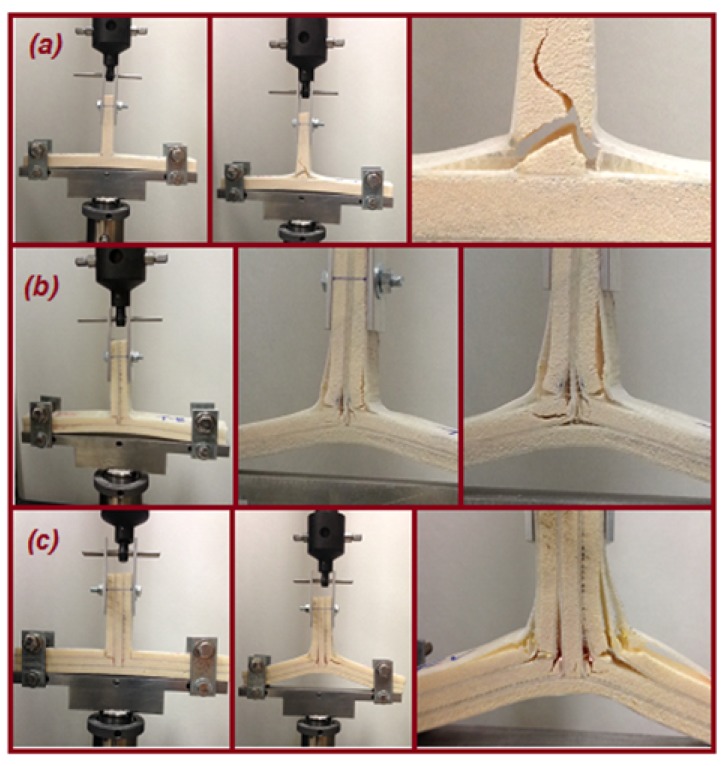
The failure sequence under pull-off loading of the T-joint. (**a**) Reference design; (**b**) biomimetic (2 × 2) design; (**c**) biomimetic v-notched (3 × 3) design.

**Figure 15:**
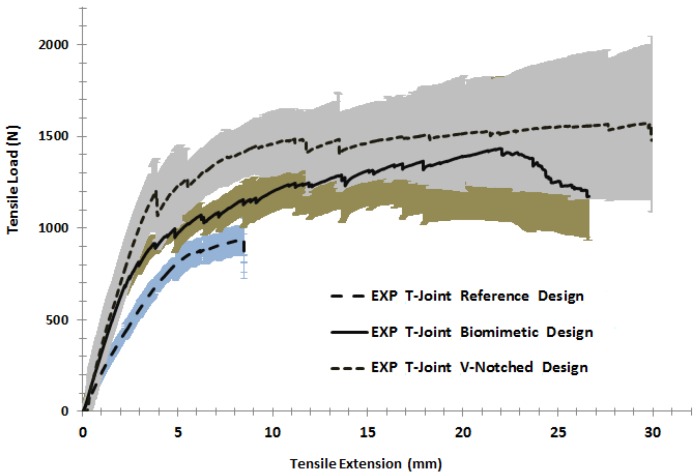
Comparisons of T-joint strength for the reference design, the biomimetic (2 × 2) design and the biomimetic v-notched (3 × 3) design under pull-off loading. The lines represent the average values, while the shaded area represents the standard deviation of the five specimens of each kind of T-joint design.

Figure 15 shows the comparison of three T-joint load response behaviors under tension load. It is obvious that the biomimetic fabricated design improved the overall strength and stiffness to postpone the final failure of the T-joint. However, the first failure was fairly less than the reference T-joint design. When the tension load increased, the reference T-joint samples started to fail in a nonlinear pattern after an extension of 5 mm as the crack initiated to cause the debonding between the fiberglass layer and the epoxy filler in both fillet regions. After that, the cracks propagated from each side of the lower web core foam to meet in the area to grow upward to the load support, causing the final failure at a load of 950 N and an extension of 8.5 mm. This indicates that the crack was caused by the shear failure mode in the web core, as in Figure 14a.

Based on the biomimetic (2 × 2) T-joint curve in Figure 15, the strength and stiffness of the sandwich T-joint was fairly increased compared to the strength of the reference T-joint design. The debonding/delamination was developed in two areas on both sides of the fillet region to cause the first failure. During the load, the cracks propagated to the core flange and the web on both curvature sides of the T-joint. Another layer separation arrested at the second fillet region in which the four fiberglass layers were combined to cause the growing of the cracks in the web, as well as the shear crack appearing in the core flange, as in Figure 14b. However, the debonding and core cracks were not enough to cause the final failure. The main reason is that the tension load was applied in the longitudinal direction of the reinforcement of the fiberglass layers. Eventually, the results show that the biomimetic fabrication increased the overall T-joint strength/stiffness in addition to gaining the reasonable delay of the T-joint final failure.

The final curve in Figure 15 shows the result of the biomimetic T-joint with the v-notched design. It reveals that the joint strength/stiffness was improved more than 1.5 times that of the strength of the reference T-joint design, while the difference of the Biomimetic (2 × 2) T-joint design was to improve the first failure event. As mentioned early, applying the v-shaped fabrication has the ability to reduce the interlaminar stress in the critical fillet region, since it distributes the applied loads precisely in that region. The first failure, therefore, of the structure reached a load of 1200 N and an extension of 3.5 mm. This was represented as debonding at both upper fillet areas when the skin separated from the fillet, followed by an interfacial crack (foam/fiberglass) propagating into the web and flange sides and the kinking crack growing into the foam core. As the load increased, another delamination between the skin layers was developed at the area of the combined skin of the longest web/core, and then, two kinking core cracks propagated through up the parallel second flange core, as in Figure 14c. It was noted that the the lowest core flange was rushed as the load increasing. The design requirements were satisfied since the v-noted T-joint was able to sustain the rising load to postpone the final failure compared to the first two cases. The results illustrate that the biomimetic v-noted fabrication has the highest strength to practically delay the final failure.

### 6.2. Biomimetic Sandwich T-Joint Efficiency

**Figure 16:**
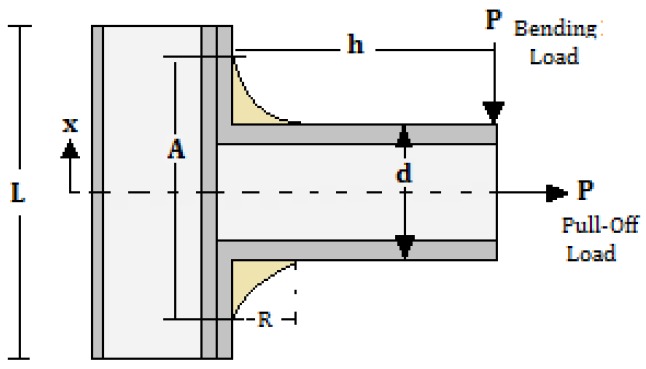
Sketch of the T-joint configuration used in the formulation of deflection in the bending and tensile loads.

To examine T-joint efficiency, the beam moment-deflection relationship is used to investigate the relative web deformation caused by flange deflection along the interface connection between the web and flange. The deformation can be determined from the deflection sustained by the flange section due the initial failure load ($P_{int}$), as in Table 5, in the bending and pull-off loads. Figure 16 shows the connection area of the web/flange, where the first initial failure occurred, which was used to calculate the deflection deformation.

The deflection of the sandwich T-joint in the region (0≤×≤d/2) was calculated in bending and tension based on Equation (9) and Equation (10) [26].

(9) $$y\left(x\right)=\frac{Px^{2}\left[6\left(h-R\right)-3d-\frac{2x^{2}}{2R+d}\right]}{48{\left(EI\right)}_{eqv}}$$

(10) $$y\left(d\right)=\frac{Pd^{2}\left[12\left(h-R\right)-2d-\left(3+\frac{d}{2R+d}\right)\right]}{348{\left(EI\right)}_{eqv}}$$

where ${\left(EI\right)}_{eqv}=E_{f}\left(bt_{f}{\left(t_{c}+2t_{f}\right)}^{2}\right)/2$ is the equivalent flexural rigidity of the bi-material flange section [27]. To normalize the data to account for the weight of each T-joint design, the measured deflection at initial failure was divided by the specimen weight, $w_{T}$, as in Table 5, resulting in the quantity deflection/weight. Accordingly, the deflection/weight was normalized to connect the weight effect on T-joint efficiency.

**Figure 17:**
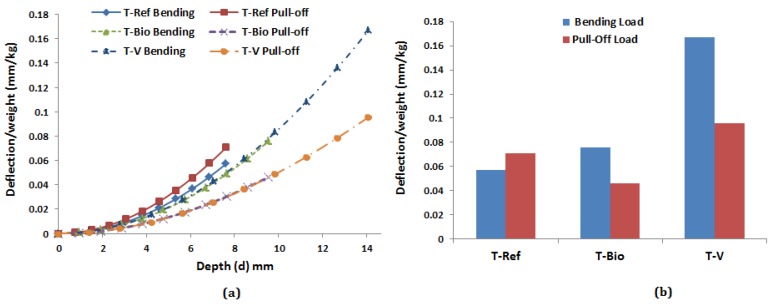
(**a**) Deflection/weight vs. the half depth of the web in each T-joint design; (**b**) deflection/weight vs. the type of T-joint for the bending and pull-off loads.

Figure 17 shows the results of the deflection/weight vs. the half depth of the web in each T-joint design at the initial failure of bending and pull-off loads. It is clear from both graphs that the results indicate that the biomimetic v-notched T-joint has the highest efficiency. Despite the fact that it has the highest weight compared to other T-joint designs, it can sustain the highest deflection before the initial failure takes place in both load cases. The deflection induced by bending load, however, is higher by about 42.7% than the pull-off load. For the reference and biomimetic (2 × 2) T-joints, the results show that the bending deflection of the biomimetic (2 × 2) T-joint improved by 24.47% compared to the reference T-joint design. However, the pull-off deflection was not improved because the initial failure load in the tension was not improved, adding to the weight effect. Eventually, it can be concluded that the biomimetic fabrication as the v-notched shape increases the joint efficiency, although the fabrication steps produce extra gained weight from the reinforcement process of epoxy/resin with fiberglass.

### 6.3. Finite Element versus Experimental Results

#### 6.3.1. Comparison of the Reference T-joint Design

**Figure 18:**
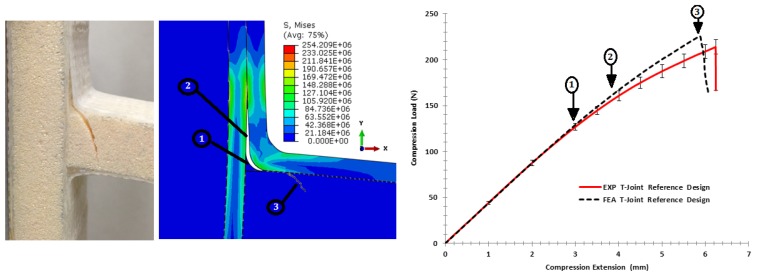
Comparison of the finite element vs. the experiment results for the reference T-joint design validation under bending load.

Figure 18 demonstrates the comparison of the strength of the reference design of the sandwich T-joint under bending load. It can be clearly seen that the stiffness responses from the FE model correlate well with the experimental measurement until an extension of 3 mm; then, the initial non-linearity response occurred. This indicates the onset of the first failure due to the interface separation at the upper curvature of the filler/skin region referred to by (1) in graph. The second debonding (2) occurred between skin/skin plies along the extension of the first interfacial crack combined with the facesheet deformation. The final failure mode (3) occurred in the web core as a single shear crack, as it initiates from the curvature area of high stress, then propagates in the center of the web core. Indeed, the place of final failure follows the trend of the experimental result. This means that the parametric interfacial material study of the mixed-mode interface model, the Hashin model of skin and the crushable models of the core in that load direction (transverse properties) are considered as a conservative estimation, as the modeling is close enough to the actual test.

**Figure 19:**
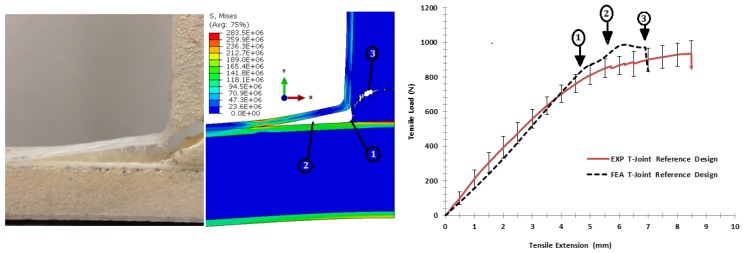
Comparison of the finite element vs. the experiment results for the reference T-joint design validation under the pull-off load.

Additionally, Figure 19 shows the strength comparison of the reference T-joint under the pull-off load. It reveals that in the elastic region of the force-displacement response, both curves are almost identical until an extension of 4 mm; then, the initial non-linearity response occurred because of the interface separation at both T-joint curvatures of the filler/skin region referred to in (1). Visual examination of the failure surfaces revealed that since the delamination becomes severe in the skin/skin interface as in (2), the bottom of the core web in between the curvature region had the highest tensile stress to cause the initiation of the shear cracks at both sides; then, the cracks propagated to meet in the center of the web, inducing sudden final fracture as in (3).

#### 6.3.2. Comparison of the Biomimetic (2 × 2) Design

**Figure 20:**
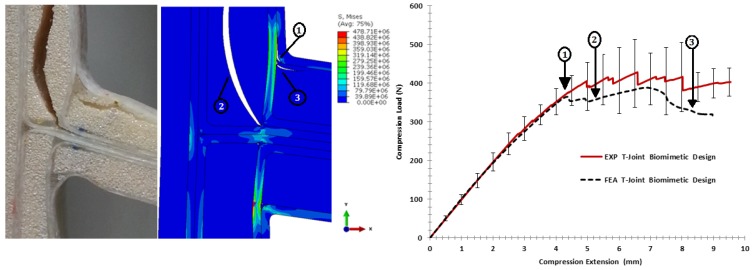
Comparison of the finite element vs. the experiment results for the biomimetic (2 × 2) design validation under the bending load.

The comparison of the strength and the failure sequence of the biomimetic (2 × 2) T-joint under bending load is shown in Figure 20. The first debond appeared at the upper curvature filler/facesheet interface at an extension of 4.4 mm, referred to as (1) in graph. Before that, the elastic stiffness response of the FE model correlated well with the experimental result. It is noted that the first failure of the FE model occurred faster than the experiment, but it is still within the experimental range. After the initial failure, the large core shear crack grew from the center of the structure similar to the experiment as in (2); then, the second cracks grew underneath the upper fillet region, as in (3). However, despite the fact that the structure integrity was slowly reduced, the joint exhibits the increasing of load extension because of the internal facesheet layers having not debonded to delay the final failure. Further, the shape of the crack failure in the FE model is similar to the experimental result, since most failure modes occurred under mixed-mode load conditions. This confirms that parametric interfacial materials in that load direction (transverse properties) are well estimated, as the modeling results of failure shapes correspond to the actual test.

**Figure 21:**
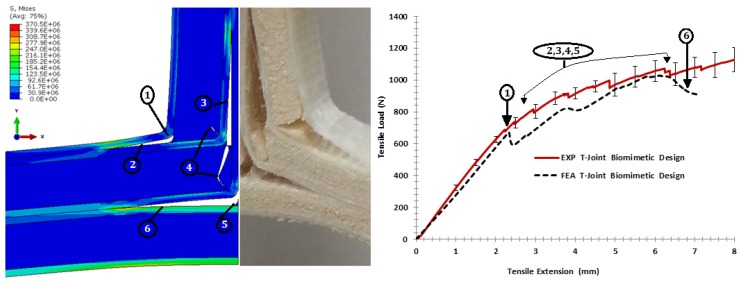
Comparison of the finite element vs. the experiment results for the biomimetic (2 × 2) design validation under the pull-off load.

Under pull-off load, Figure 21 shows the fracture comparison of the biomimetic (2 × 2) T-joint. It reveals that the elastic region of curves is almost similar until an extension of 2.2 mm; then, the initial failure occurred because of the interface separation at both T-joint curvatures of the filler/skin region, as in (1). When the load was increased, the failure surfaces revealed multi-delamination at the skin/skin interface in different places as in (2), (3), (5) and (6); then, the core cracks grew corresponding to the experiment failure events, as in (4). In general, the interfacial material parameters of the FE model were fairly consistent in that load direction (longitudinal properties), as the model described most experimental failure events.

#### 6.3.3. Comparison of the Biomimetic V-Notched (3 × 3) Design

**Figure 22:**
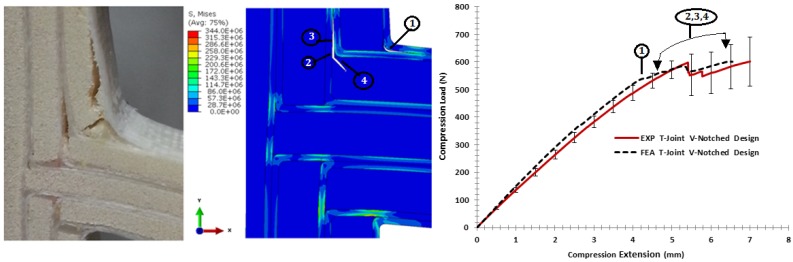
Comparison of the finite element vs. the experiment results for the biomimetic v-notched design validation under the bending load.

Figure 22 shows the variation of the fracture scenarios for the biomimetic v-notched sandwich T-joint under the bending load. The stiffness of FE modeling lies in the range of the elastic stiffness experimental measurement. After an extension of about 4 mm, the onset of failure is indicated as the initial non-linearity response. The combination of the interface separation at the upper curvature of the filler/skin region, as in (1), and facesheet deformation had a major effect to reduce the structural integrity. When the load increased, the second debond grew in the interface of the filler/skin in the area parallel to the load direction, as in (2); then, the interfacial crack extend to the third debonding between the skin/skin, as in (3). In the same area, different failure modes initiated in the web core as a single shear crack, then propagated to the center of the web core, as in (4). It is necessary to note that the FE model has difficulty to converging after this extension, even though the solution method was modified with the line search algorithm. The main reason for that is that the model has a large number of contacts, and multi-fracture criteria are involved. However, the shapes of the crack failure in the FE model are similar to the initial phase of the experimental result, as they are initiated and propagated under mixed-mode load conditions. This emphasizes the good estimation of the interface and fracture parameters, as well as the parametric materials, which can handle the FE modeling to give the failure shapes corresponding to the actual test.

**Figure 23:**
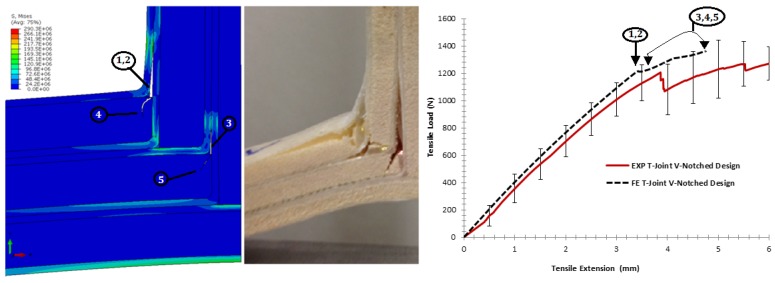
Comparison of the finite element vs. the experiment results for the biomimetic v-notched design validation under the pull-off load.

For the pull-off load case, Figure 23 shows the strength comparison of the biomimetic v-notched T-joint. The elastic region of the force-displacement curve of the FE model was close enough to the experiment until an extension of 3.5 mm. After that point, the initial debonding appeared in the interface of the filler/skin region at both T-joint curvatures, as in (1). The failure surface of the FE results, then, show that the interface crack grew up to the skin/skin interface, as in (2), to induce high tensile stress in the first lower core flange to cause kinking crack initiation, as in (4), then grew as a shear crack as the experiment crack shape. During the load increase, the second delamination appeared in the interface of the skin/skin in the middle of the second layers of the biomimetic T-joint, as in (3), to cause the crack kinking into the second flange of the core, as in (5). Again, the difficulty of convergence restricts the solution to the current extension of (5 mm). The reason for that is that the contact and failure criteria cause high perturbation, as the increment of load history increased. However, the initial assessment of the fracture and material parameters is in good shape, since the FE solution is following the trend of the experiment.

### 6.4. Stiffness Evaluation of Biomimetic T-Joint Designs

**Figure 24:**
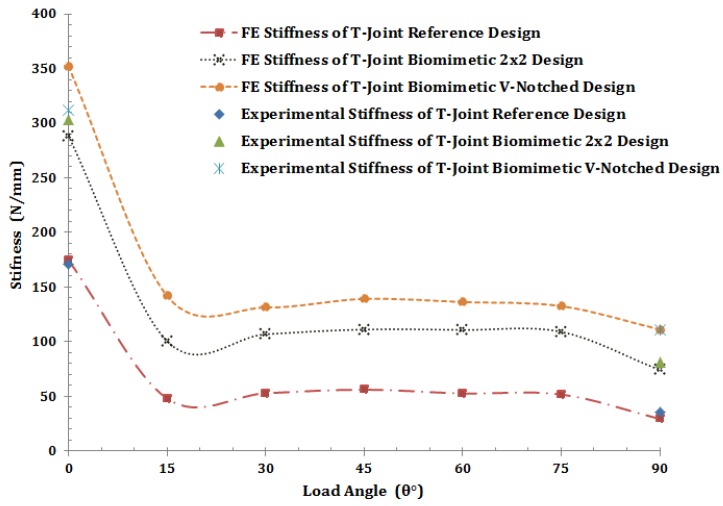
Experimental and finite element stiffness vs. the load angle variation.

The main reason for the study of the stiffness index at the border of the first failure initiation is to investigate how much the sandwich T-joint designs gain stiffness and strength under the hypothesis of biomimetic fabrication. The FE models are used to cover the load angle range between 0° and 90°. This also can examine the comprehensive failure range of the T-joint structure under the biomimetic design in terms of the stiffness investigation. Figure 24 shows the stiffness variations of three T-joint designs versus different angles of applied load. It reveals that the stiffness of the biomimetic (2 × 2) T-joint is increased by about 50% of the stiffness of the reference T-joint design for the range of load angles. However, the highest gain of stiffness is recorded in the biomimetic v-notched design to reach about 22% of the biomimetic (2 × 2) T-joint stiffness. The graph also shows the experimental stiffness at angles of 0° and 90° in all three T-joint designs, which are correlated consistently with the FE model results. It is noted that the stiffness rate of each T-joint design in the range of the angle of the load from 15° to 75° has small variations, while the highest values are recorded at an angle load of 0° and the lowest value at 90°. This means that the bending stiffness (*D*) of each T-joint design in the range of 15°≤D≤90° has no significant change in the first failure case. However, the bending stiffness increases significantly when the biomimetic hypothesis takes place in the fabrication of the T-joint design.

## 7. Conclusions

Composite sandwich T-joints with biomimetic architectures under tensile and bending loads were investigated analytically and experimentally. It was concluded that the overall T-joint stiffness and strength were significantly enhanced. The results reveal that the cause of initial failure was the critical location in the corner of the joint at the skin/filler adhesive interface. The biomimetic designs were able to improve the initial failure limit in the case of bending loads, whereas the initial failure limit was reduced in the case of the tension load, especially in the biomimetic (2 × 2) T-joint. Additionally, it was noted that the core kinking crack failure and delamination failure modes were dominant defects. Moreover, the joint efficiencies related to the weight and deflection of the three sandwich composite T-joint designs at the first failure loads were discussed. It was shown that the efficiency of T-joints significantly improved using the biomimetic approach, such as the v-notched shape. The fabrication steps of this shape produced extra weight from the reinforcement process of epoxy/resin with fiberglass.

As the summary of the numerical validations, the finite element (FE) model accurately predicted the stiffness, strength and fracture modes of the biomimetic composite sandwich T-joint under bending and pull-off loading. The combination of the mixed mode cohesive element and other failure criteria was used in the modeling. The FE model explored the initiation and propagation of defects until the complete failure load history. The results indicated that the onset of failure was in agreement consistently with the experimental observations. The interface (fiber/filler) layers in the curvature region were the most critical location for the onset of delamination in all modeling of the three T-joint designs. Moreover, the FE model was able to capture most of the multiple debonding events and kinking core crack scenarios. Strengthening and delamination processes of multi-interfaces were investigated in terms of adjusting the mixed-mode key parameters of multi-interface materials corresponding to experimental results.

## Figures and Tables

**Table 1:** T-joint dimensions and lay-up orientations.

T-Joint	Dimensions				Skin Ply	Foam	Skin
	*L*	*h*	*W*	Radius	Thickness	Thickness	Layup
Configuration	(mm)	(mm)	(mm)	(mm)	(mm)	(mm)	*θ*
Reference	228	72.5	25.4	1	1	12.7	0°
Biomimetic (2 × 2)	228	72.5	25.4	1	1	6.35	0°
Biomimetic v-notched	228	72.5	25.4	1	1	6.35	0°

**Table 2:** Elastic properties of the T-joint bulk materials.

Materials	E${}_{11}$	E${}_{22}$	E${}_{33}$	G${}_{12}$	G${}_{13}$	G${}_{23}$	v${}_{12}$	v${}_{13}$	v${}_{23}$
	(GPa)	(GPa)	(GPa)	(GPa)	(GPa)	(GPa)			
Unidirectional Fiberglass S-1/Epoxy [19]	29.26	11.32	11.32	2.79	2.79	2.61	0.175	0.175	0.34
Foam Divinycell H80 [15]	0.085	-	-	0.0322	-	-	0.32	-	-
Filler (Epoxy of EPON 828/EPI-CURE 3223) [20]	3	-	-	1.136	-	-	0.32	-	-

**Table 3:** Summary of the failure modes of S-1 glass.

G${}_{ft}^{c}$	G${}_{fc}^{c}$	G${}_{mt}^{c}$	G${}_{mc}^{c}$
(J/m${}^{2}$)	(J/m${}^{2}$)	(J/m${}^{2}$)	(J/m${}^{2}$)
4288	4288	4100	4100

**Table 4:** T-joint interface materials.

Interface Materials	*σ*°	*τ*°	$K_{I}$	$G_{IC}$	$G_{IIC}$	*η*
	(MPa)	(MPa)	(Pa/m)	(J/m${}^{2}$)	(J/m${}^{2}$)	
Fiber/Fiber	36.25	32.5	$1.42\times 10^{14}$	1037±431 [25]	1276±555 [25]	2.45
Fiber/Filler	72.5±23.5 [20]	102.5	$1.5\times 10^{14}$	306	572	4.4
Foam/Filler	3.36	7.32	$1.25\times 10^{14}$	306	572	2.284
Fiber/Foam${}_{tc=12.7mm}$ [19]	3.3	3.3	$1.42\times 10^{12}$	496	661	4.35
Fiber/Foam${}_{tc=6.35mm}$ [19]	2.97	2.97	$1.42\times 10^{12}$	378	504	2.35

**Table 5:** Experimental results summary.

T-Joint Specimen ID	Bending Load		Pull-Off Load		Weight	Depth
	P${}_{int}$ (N)	P${}_{max}$ (N)	P${}_{int}$ (N)	P${}_{max}$ (N)	w${}_{T}$ (g)	d (mm)
Reference Design	185.8 ± 6.9	185.8 ± 6.9	860.1 ± 54.2	881.7 ± 75.6	44.4 ± 1.75	15.24 ± 0.08
Biomimetic (2 × 2)	390.8 ± 62.3	428.2 ± 87.9	680.2 ± 32.6	1413.1 ± 372.8	83.8 ± 3.24	19.1 ± 0.17
Biomimetic V-Notched	598.2 ± 37.3	603.3 ± 89.3	1206.5 ± 172.7	1572.9 ± 426.9	103.7 ± 3.69	28.15 ± 0.9

## References

[1] Bozhevolnaya E., Lyckegaard A., Thomsen O.T. (2008). Novel design of foam core junctions in sandwich panels. Compos. Part B: Eng..

[2] Toftegaard H., Lystrup A. (2005). Design and test of lightweight sandwich T-joint for naval ships. Compos. Part A: Appl. Sci. Manuf..

[3] Diler E.A., Özes Ç., Neşer G. (2009). Effect of T-joint geometry on the performance of a GRP/PVC sandwich system subjected to tension. J. Reinforced Plast. Compos..

[4] Khalili S.M.R., Ghaznavi A. (2011). Numerical analysis of adhesively bonded T-joints with structural sandwiches and study of design parameters. Int. J. Adhes. Adhes..

[5] Shenoi R.A., Read P.J.C.L., Jackson C.L. (1998). Influence of joint geometry and load regimes on sandwich tee joint behavior. J. Reinforced Plast. Compos..

[6] Theotokoglou E.E. (1997). Strength of composite T-joints under pull-out loads. J. Reinforced Plast. Compos..

[7] Dharmawan F., Thomson R.S., Li H., Herszberg I., Gellert E. (2004). Geometry and Damage Effects in A Composite Marine T-Joint. Compos. Struct..

[8] Nanayakkara A.M., Feih S., Mouritz A.P. (2013). Improving the fracture resistance of sandwich composite T-joints by z-pinning. Compos. Struct..

[9] Bianchi F., Koh T.M., Zhang X., Partridge I.K., Mouritz A.P. (2012). Finite element modelling of z-pinned composite T-joints. Compos. Sci. Technol..

[10] Burns L.A., Mouritz A.P., Pook D., Feih S. (2012). Bio-inspired design of aerospace composite joints for improved damage tolerance. Compos. Struct..

[11] Thummalapalli V.K., Donaldson S.L. (2012). Biomimetic Composite Structural T-joints. J. Bionic Eng..

[12] Mattheck C., Bethge K. (1998). The structural optimization of trees. Naturwissenschaften.

[13] Burns L.A., Mouritz A.P., Pook D., Feih S. (2012). Strength improvement to composite T-joints under bending through bio-inspired design. Compos. Part A: Appl. Sci. Manuf..

[14] Burns L.A., Pook D. Tree joints: Biomimetic insights for aerospace composite joints. Proceedings of the 27th International Congress of the Aeronautical Sciences.

[15] Divinycell H. Fiberglass Supply H. GRADE. DIVINYCELL^®^. *Technical Manual* Manual-11.99-1.

[16] AGY (2013). Data Sheet of S-1 HM^TM^ Glass Fibers. Technical Manual.

[17] Momentive (2005). Data Sheets of EPON resin 828 and EPIKURE 3223 curing agent. Technical Manual.

[18] Saeid A.A., Donaldson S.L. Characterization and Simulation of Divinycell H80 Closed-Cell Foam. Proceedings of the American Society of Composites-30th Technical Conference.

[19] Saeid A.A., Donaldson S.L. Facesheet to Core Interface Characterization of Sandwich Structure. Proceedings of the American Society of Composites-30th Technical Conference.

[20] (2001). EPON Resin Structural reference manual-curing agent (Diethylenetriamine EPI-CURE 3223) systems, *Technical Manual* Appendix 1.

[21] (2013). ABAQUS/CAE Version, A. 6.13 Documentation (Abaqus). Abaqus user’s guide. *User’s Manual 24.3.3*.

[22] Cui W.C., Wisnom M.R., Jones M. (1992). A comparison of failure criteria to predict delamination of unidirectional glass/epoxy specimens waisted through the thickness. Composites.

[23] Camanho P.P., Dávila C.G. (2002). Mixed-Mode Decohesion Finite Elements for the Simulation of Delamination in Composite Materials.

[24] Camanho P.P., Davila C.G., de Moura M.F. (2003). Numerical simulation of mixed-mode progressive delamination in composite materials. J. Compos. Mater..

[25] Alessa H.A. (2014). Delamination in Hybrid Carbon/Glass Fiber Composites. Ph.D. Thesis.

[26] Tang J.H., Sridhar I., Chai G.B., Ong C.H. (2013). Modelling of Composite Sandwich T—Joints Under Tension and Bending. Advances in Modeling and Design of Adhesively Bonded Systems.

[27] Gryzagoridis J., Oliver G., Findeis D. (2015). On the equivalent flexural rigidity of sandwich composite panels. Insight-Non-Destr. Test. Cond. Monit..

